# Association of alcohol use and multimorbidity among adults aged 40 years and above in rural South Africa

**DOI:** 10.1038/s41598-023-35018-6

**Published:** 2023-05-14

**Authors:** Mafuno G. Mpinganjira, Tobias Chirwa, Chodziwadziwa. W. Kabudula, Francesc Xavier Gómez-Olivé, Stephen Tollman, Joel Msafiri Francis

**Affiliations:** 1 Department of Family Medicine and Primary Care, Faculty of Health Sciences, School of Clinical Medicine, University of the Witwatersrand, Johannesburg, South Africa; 2 Division of Epidemiology and Biostatistics, Faculty of Health Sciences, School of Public Health, University of the Witwatersrand, Johannesburg, South Africa; 3 MRC/Wits Rural Public Health and Health Transitions Research Unit (Agincourt), Faculty of Health Sciences, School of Public Health, University of the Witwatersrand, Johannesburg, South Africa

**Keywords:** Diseases, Medical research, Risk factors

## Abstract

We assessed the prevalence of reported alcohol use and its association with multimorbidity among adults aged 40 years and above in a rural, transitioning South African setting. Findings could potentially inform alcohol interventions integration in the prevention and treatment of chronic conditions. We analysed data from the first wave of The Health and Ageing in Africa—a longitudinal Study in an INDEPTH community (HAALSI) nested within the Agincourt Health and Demographic Surveillance Systems, conducted between November 2014 and November 2015 (n = 5059). We computed descriptive statistics and performed univariate analysis to determine factors independently associated with multimorbidity. Age, Body Mass Index, education, sex, and household wealth status and variables with a *p*-value < 0.20 in univariate analysis were included in multivariable Modified Poisson regression models. Any factors with a *p*-value of < 0.05 in the final models were considered statistically significant. The first wave of HAALSI was completed by 5059 participants aged 40 years and above and included 2714 (53.6%) females. The prevalence of reported ever alcohol use was 44.6% (n = 2253) and of these 51.9% (n = 1171) reported alcohol use in the last 30 days. The prevalence of HIV multimorbidity was 59.6% (3014/5059) and for multimorbidity without HIV 52.5% (2657/5059). Alcohol use was associated with HIV multimorbidity among all participants (RR: 1.05, 95% CI: 1.02–1.08), and separately for males (RR: 1.05, 95% CI: 1.00–1.10) and females (RR: 1.06, 95%CI: 1.02–1.11). Similarly, alcohol use was associated with multimorbidity without HIV among all participants (RR: 1.05, 95% CI: 1.02–1.09), and separately for males (RR: 1.06, 95% CI: 1.00–1.12) and females (RR: 1.06, 95% CI: 1.01–1.11). Reported alcohol use was common and associated with HIV multimorbidity and multimorbidity without HIV among older adults in rural northeast South Africa. There is a need to integrate Screening, Brief Interventions, and Referral for alcohol Treatment in the existing prevention and treatment of multimorbidity in South Africa.

## Introduction

Harmful alcohol use is a significant public health problem globally^1,2^. Excessive alcohol use (defined as the act of binge drinking that includes ≥ 4 drinks at once for women and ≥ 5 drinks at once for men)^3^ has direct impact on health-related Sustainable Development Goal 3 (SDG 3) through infectious diseases (for example HIV^2,4,5^, TB, and viral hepatitis), Non Communicable Diseases (NCDs), and mental health. In 2016, approximately 49% of alcohol attributable Disability-adjusted life years (DALYs) were due to NCDs and mental health conditions. Moreover excessive alcohol use resulted in 1.7 million deaths from NCDs, while 12.9% deaths attributable to alcohol consumption were due to infectious diseases^2^.

Excessive alcohol use is increasing in Sub-Saharan Africa (SSA), resulting in substantial cognitive, behavioural, and physiological symptoms. Previous studies indicate that approximately 20% of all individuals attending healthcare facilities had alcohol use disorder (AUD)^6–8^. Despite the magnitude of this impact, Mushi et al.^9^ found that only less than 1% of those with AUD were diagnosed and received appropriate treatment. Furthermore, a report by the World Health Organisation (WHO) showed that strategies to prevent excessive alcohol use and further interventions were scarce in SSA^2^ as such there is no integration of alcohol use interventions like Screening, Brief Intervention and Referral for Treatment (SBIRT) in either primary health care (PHC) or in the management of multimorbidity^9,10^.

Multimorbidity is an escalating public health problem that poses significant impact on quality of life and resulting in increased health threats and financial burden to health systems and populations^11–13^. Alcohol use is one of the four major risk factors for multimorbidity^11,13,14^. The main clinical complications associated with excessive alcohol use include HIV, hypertension, diabetes, mental health, and liver fibrosis and cirrhosis^15–24^. This occurs mainly due to the toxic biochemical effects of alcohol that may increase the risk of organ damage, compromise treatment effectiveness, or even the safety of prescribed medications due to chemical interactions^25^.

Both excessive alcohol use and multimorbidity are recognised as significant problems in SSA^2^ but their association has not been properly studied as well as their impact in rural African settings. This paper reports on the prevalence and association between reported alcohol use and multimorbidity (with and without HIV) among older adults aged ≥ 40 years in a rural South African setting. The study findings could potentially inform targeted alcohol reduction interventions amongst those most at risk, integration in multimorbidity prevention and treatment at PHC and community levels.

## Methods

### Study design, setting, and sample

The study used data from the baseline wave of the Health and Ageing in Africa—a longitudinal Study in an INDEPTH community (HAALSI)^26^. In brief, HAALSI is a longitudinal cohort study which recruited individuals aged ≥ 40 years who are enrolled in the Agincourt Health and Demographic Surveillance Systems (HDSS) and resident in the Bushbuckridge subdistrict of rural Mpumalanga, northeast South Africa. HAALSI aims to describe biological, social and economic determinants and consequences of health and ageing in rural South Africa^26^.The Agincourt HDSS, which is hosted by the South African Medical Research Council/Wits University Rural Public Health and Health Transitions Research Unit, has since 1992 collected longitudinal population-level data on vital demographic events (births, deaths, in-migration, and out-migrations) and other key health, social and economic indicators in the Agincourt study site^26^. Out of a total number of 12,875 eligible individuals from the Agincourt HDSS population, 6281 were randomly selected to participate in the HAALSI study, and 5059 (80.5%) completed the baseline wave. Data from all 5059 enrolled participants were analysed in our study.

### Study visits

Collection of data for the baseline wave of the HAALSI study took place between November 2014 and November 2015. Trained fieldworkers visited participants in their homes and collected data on sociodemographic variables and self- reported health status and risk factors using Computer-Assisted Personal Interviews (CAPI) and performed clinical assessments including blood pressure and point-of-care biomarkers. Dried blood spots were also collected for assessment of HIV serostatus and viral load. The survey instruments were translated from English into xi-Tsonga, the local language, and responses were back translated into English to ensure reliability. Translation was performed by experienced members of the unit staff with further minor modifications by the fieldworkers who conducted the interviews to ensure the language used was in keeping with the vernacular^26^.

### Study variables

#### Sociodemographic variables

Sociodemographic variables were self- reported and included age, marital status, employment status, educational attainment, marital status, number of individuals living in household, and Principal Component Analysis(PCA) was used to create a wealth index from household characteristics and asset ownership^27^).

#### Chronic illnesses and measurements

Body Mass Index (BMI) was calculated from objective measures of weight (in kg) divided by square height (in meters) and WHO BMI categories were used^28^.

A total of 8 chronic illnesses were included in our study and defined by both objective measures^29^ and self-report. Presumptive diagnosis of hypertension, diabetes, and dyslipidaemia was through the existing self-report of the diagnosis of the conditions. Additionally, objective measures were used to verify this: hypertension—mean systolic blood pressure ≥ 140 mmHg or mean diastolic blood pressure ≥ 90 mmHg—was calculated from the second and third of three consecutive measurements during the home visit^30^ for those not on hypertension treatment. Those on hypertension treatment were classified as hypertensives regardless of the blood pressure measurement results at a home visit; diabetes mellitus—fasting glucose ≥ 7 mmol/L or random glucose ≥ 11.1 mmol/L on point-of-care testing during the home visit (CareSens N monitor; i-SENS, Seoul, South Korea)^31^ and dyslipidaemia—total cholesterol > 6.21 mmol/L, high-density lipoprotein < 1.19 mmol/L, low-density lipoprotein > 4.1 mmol/L or triglycerides > 2.25 mmol/L on point-of-care testing (CardioChek PA; PTS Diagnostics, Whitestown, Indiana, USA)^29^. Anaemia was defined as haemoglobin < 12 g/dL in women and < 13 g/dL in men on point-of-care testing (Hemocue Hb201 + analyser; Haemocue, Sweden)^32^. Individuals were considered HIV positive if dried blood spots were positive on screening (Vironostika Uniform 11; Biomeriuex, France) and subsequent confirmatory tests (Roche Elecsys; Roche, USA). Dried blood spots from individuals who tested positive for HIV were then tested for HIV-1 RNA (BioMérieux NucliSens; lower limit of detection 100 copies/mL). Angina was defined using the Rose criteria^33^ and chronic bronchitis was defined as a self-reported daily cough, productive of phlegm, for at least 3 months per year for at least 2 successive years^34^. Participants were classified as having depression if they identified three or more symptoms on the Centre for Epidemiological Studies-Depression (CES-D) Scale^35^, while post-traumatic stress disorder was defined as a score ≥ 4 on the Breslau Scale^36^.

#### Multimorbidity with HIV and multimorbidity without HIV

About a third of the participants in this study were living with HIV, as such in this study and the previous studies from the same cohort have classified multimorbidity into HIV multimorbidity and multimorbidity without HIV^37^. participants were considered to have HIV multimorbidity if they had two or more of these chronic illnesses including HIV^29,38,39^.We further classified the subset of individuals who presented more than one of the listed conditions but did not have HIV as one of their chronic conditions, as having multimorbidity without HIV. The chronic illnesses included were selected to ensure comparability with Health and Retirement Survey sister studies as well as to obtain further data on conditions which are prevalent in the Agincourt HDSS study area^26^.

### Statistical analysis

**Figure 1:**
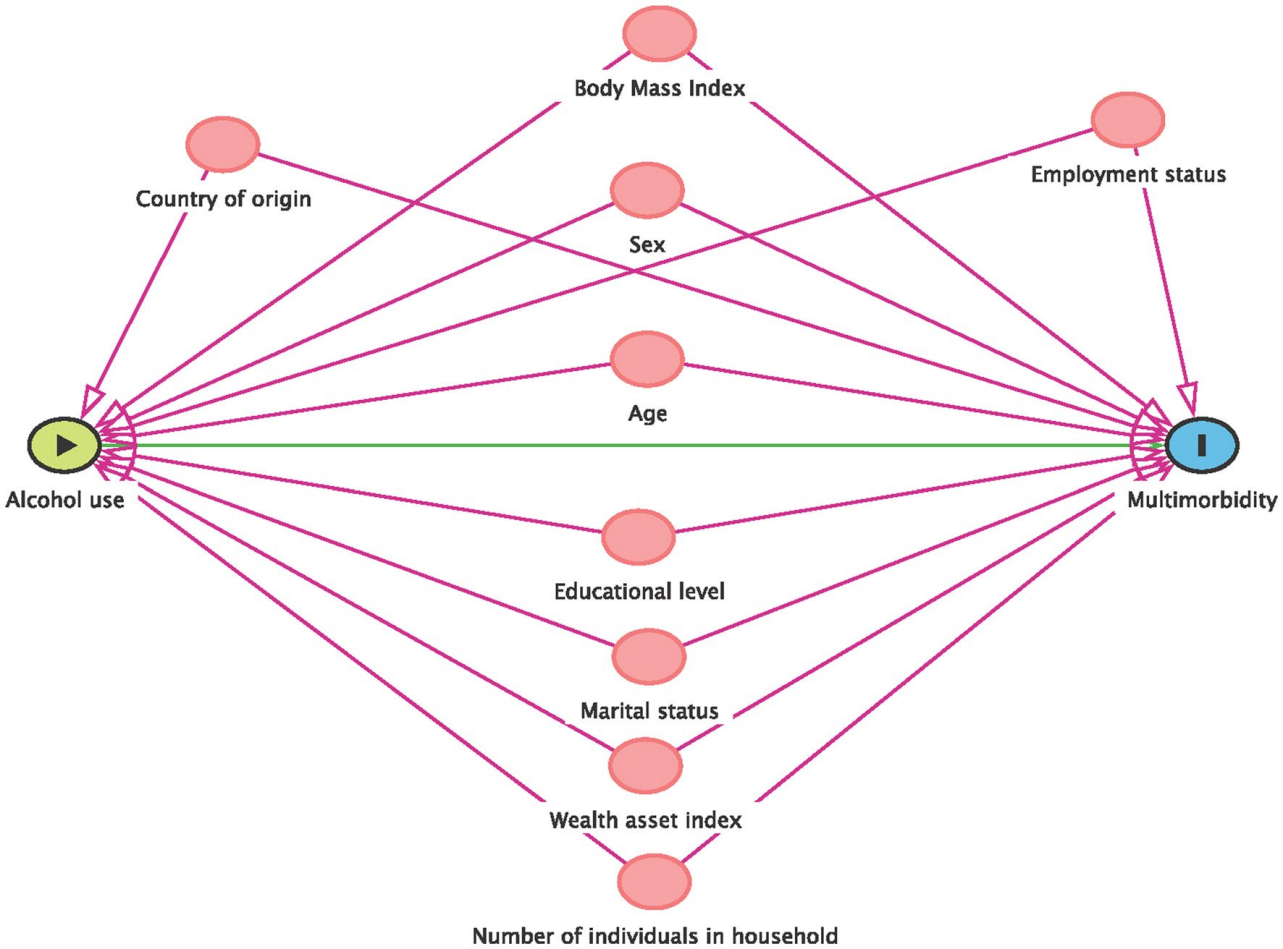
Direct acyclic graph of causal pathways for alcohol use and multimorbidity.

Data management pre-processing and analysis was conducted using STATA v17.0 (StataCorp, USA). The continuous variables (age and BMI) were categorised. The BMI was categorised according to WHO classification^28^, and all analyses were stratified by sex. Descriptive statistics were computed and reported as frequencies and proportions—this was done for sociodemographic factors, alcohol use patterns, and the prevalence of chronic conditions (both multimorbidity with HIV and multimorbidity without HIV patterns). The *Chi*^*2*^ test was used to assess the strength of the association between individual sociodemographic and household factors and multimorbidity. Age, Body Mass Index, Wealth asset index and Educational attainment were considered as a priori confounders (for male and female models) and sex^40^ (for all participants model)^41^ and, therefore, added in the multivariable modified Poisson regression models regardless of the univariate *p*-values. All other variables were entered in the multivariable models if they had a *p*-value < 0.20 from the univariate analyses (Supplementary Tables 1–6). Furthermore, we have reported the Direct Acyclic Graph (DAG) on causal associations of alcohol use and multimorbidity (Fig. 1). We reported adjusted relative risk (RR) and their corresponding 95% confidence intervals (CI). Any factor with a *p*-value < 0.05 was considered statistically significantly associated with the outcomes of interest (multimorbidity with HIV or Multimorbidity without HIV).

### Ethical considerations

The study received ethical approvals from the University of the Witwatersrand Human Research Ethics Committee (ref. M141159), the Harvard T.H. Chan School of Public Health, Office of Human Research Administration (ref. C13–1608–02) and the Mpumalanga Provincial Research and Ethics Committee. Field staff received special training on ensuring participants understood the study before providing consent.

## Results

### Study population characteristics

The HAALSI study randomly selected 6281 participants 40 years and older from the Agincourt HDSS. A total of 5059 (80.5%) were reachable, available, consented to take part, and were enrolled in the baseline wave of the study. From them 2345 were male (46.4%) and 2714 were female (53.6%). The more frequent age group was between the ages of 50–59 years old (n = 1410, 27.9%), a third had a normal BMI (n = 1719, 36.7%), and three quarters were not working (n = 3719, 73.7%). Almost half of them had no formal education (n = 2306, 45.7%) and lived in a 3–6-person household (n = 2438, 48.2%). A fifth of them belonged to households with the poorest wealth status (n = 1046, 20.7%) (Table 1).

**Table 1:** Sociodemographic factors of the HAALSI population (N = 5059), enrolled between November 2014 and November 2015.

Characteristic	Categories	Sex				Total^a^	
		Male		Female			
		n	%	n	%	n	%
Age	40–49	418	17.8	500	18.4	918	18.2
	50–59	624	26.6	786	29.0	1410	27.9
	60–69	643	27.4	661	24.4	1304	25.8
	70–79	446	19.0	432	15.9	878	17.4
	80+	214	9.1	335	12.3	549	10.9
	Total	2345	100	2714	100	5059	100
Body Mass Index	Underweight	188	8.7	70	2.8	258	5.5
	Normal	1019	47.2	700	27.7	1719	36.7
	Overweight	611	28.3	717	28.3	1328	28.3
	Obese	341	15.8	1043	41.2	1384	29.5
	Total	2159	100	2530	100	4689	100
Employment Status	Employed (part or full-time)	443	19.0	362	13.4	805	16.0
	Not working	1709	73	2010	74.3	3719	73.7
	Homemaker	186	8	335	12.4	521	10.3
	Total	2338	100	2707	100	5045	100
Educational Level	No formal education	957	40.9	1349	49.9	2306	45.7
	Some primary (1–7 years)	833	35.6	883	32.7	1716	34
	Some secondary (8–11 years)	314	13.4	260	9.6	574	11.4
	Secondary or more (12 years or more)	234	10	212	7.8	446	8.9
	Total	2338	100	2704	100	5042	100
Marital status	Never married	166	7.1	124	4.6	290	5.7
	Separated/divorced	300	12.8	350	12.9	650	12.9
	Widowed	276	11.8	1264	46.6	1540	30.5
	Currently married	1602	68.3	973	35.9	2575	51
	Total	2334	100	2711	100	5055	100
Number of individuals living in household	Living alone	330	14.1	204	7.5	534	10.6
	Living with one other	257	11	281	10.4	538	10.6
	Living in 3–6-person household	1055	45	1383	51	2438	48.2
	Living in 7+ household	703	30	846	31.2	1549	30.6
	Total	2345	100	2714	100	5059	100
Wealth asset index	Poorest	502	21.4	544	20	1046	20.7
	2	455	19.4	546	20.1	1001	19.8
	3	450	19.2	541	19.9	991	19.6
	4	457	19.5	550	20.3	1007	19.9
	Richest	481	20.5	533	19.6	1014	20
	Total	2345	100	2714	100	5059	100
Country of origin	South Africa	1663	70.9	1865	68.8	3528	69.8
	Mozambique/Other	682	29.1	844	31.2	1526	30.2
	Total	2345	100	2709	100	5054	100

^a^Total of males and females combined.

### Prevalence of reported alcohol use and multimorbidity

Almost half of the participants (n = 2253, 44.6%) reported ever used alcohol, with half of them reporting alcohol use in the last 30 days (n = 1171, 51.9%). Amongst those who reported alcohol use in the last 30 days the most frequent group were those who consumed alcohol at least once a week (n = 619, 52.9%), while the rest consumed it at least once a month.

The overall prevalence of multimorbidity with HIV was 59.6% (n = 3014) and was similar in both males and females. The prevalence of multimorbidity without HIV was 52.5% (n = 2657) and was similar in males and females (Table 2).

**Table 2:** Alcohol use patterns, multimorbidity with and without HIV patterns amongst participants of the HAALSI study (N = 5059), enrolled between November 2014 and November 2015.

Characteristic	Categories	Sex				Total^a^	
		Male		Female			
		n	%^b^	n	%^b^	n	%^b^
Ever	No	771	32.9	2032	74.9	2803	55.4
	Yes	1572	67.1	681	25.1	2253	44.6
	Total	2343	100	2713	100	5056	100
Last 30 days	No	660	42	424	62.1	1084	48.1
	Yes	912	58	259	37.9	1171	51.9
	Total	1572	100	683	100	2255	100
Frequency of consumption (among those who reported alcohol use in last 30 days)	At least once a week	519	57	100	38.6	619	52.9
	At least once a month	392	43	159	61.4	551	47.1
	Total	911	100	259	100	1170	100
Multimorbidity with HIV^c^	No chronic conditions	305	13	261	9.6	566	11.2
	1 chronic condition	728	31	751	27.7	1479	29.2
	2 or more chronic conditions	1312	56	1702	62.7	3014	59.6
	Total	2345	100	2714	100	5059	100
Multimorbidity without HIV^c^	No chronic conditions	384	16.4	325	12	709	14
	1 chronic condition	827	35.3	866	31.9	1693	33.5
	2 or more chronic conditions	1134	48.4	1523	1523	2657	52.5
	Total	2345	100	2714	100	5059	100

^a^Total of males and females combined.

^b^Column percentage.

^c^Chronic conditions (Hypertension, Diabetes, Dyslipidaemia, Angina, Anaemia, Depression, Post-traumatic Stress Disorder).

### Alcohol use and HIV multimorbidity

Reported alcohol use was associated with HIV multimorbidity. Specifically, those reporting ever using alcohol had 5% increased risk of HIV multimorbidity (RR: 1.05, 95% CI: 1.02–1.08), compared to those who had never used alcohol before. This was similar in males (RR: 1.05, 95%CI: 1.00–1.10) and females (RR: 1.06, 95%CI: 1.02–1.11). The reported current alcohol use was associated with 3% lower risk of HIV multimorbidity among all participants (RR: 0.97, 95% CI: 0.94–1.01) and among females (RR: 0.97, 95%CI: 0.91–1.04) and 2% lower risk of HIV multimorbidity (RR: 0.98, 95% CI: 0.94–1.03) in males but these were not statistically significant.

### Other factors associated with HIV multimorbidity

#### Among all participants

Other factors that were significantly associated with HIV multimorbidity among all participants were BMI—specifically, the overweight category had a 10% higher risk (RR: 1.10, 95% CI: 1.05–1.16) and the obese category had a 12% higher risk (RR:1.12, 95%CI:1.09–1.16) compared to the normal weight category; marital status—especially in those reporting being separated or divorced (RR: 1.13, 95% CI: 1.05–1.22) and widowed (RR: 1.13, 95% CI: 1.05–1.21) in comparison to those who had never been married before. On the contrary, the following factors were found to be protective against HIV multimorbidity: education—specifically those who reporting completed secondary education or more had an 8% lower risk (RR: 0.92, 95% CI: 0.87–0.98) when compared to those who had no formal education; and individuals living in a 3–6-person household who had a 5% lower risk (RR: 0.95, 95% CI: 0.91–0.99) compared to those living alone.

#### Among females

Among females, other factors that were significantly associated with HIV multimorbidity were: marital status—those reporting being separated or divorced had a 14% higher risk (RR: 1.14, 95% CI: 1.03–1.25), whereas educational level had protective effect with those who completed secondary level or more having a 10% lower risk (RR: 0.90, 95% CI: 0.83–0.98).

#### Among males

Among males, factors that were significantly associated with HIV multimorbidity were: BMI—those in the overweight and obese categories had 10% higher risk (RR: 1.10, 95% CI: 1.05–1.16) and 15% (RR: 1.15, 95%CI:1.09–1.21) respectively compared to normal weight category; marital status—those who were widowed had a 19% higher risk (RR: 1.19, 95% CI: 1.05–1.34) and those who reporting currently married had a 14% higher risk (RR: 1.14, 95% CI: 1.02–1.27); and wealth index—those in the richest category (RR: 1.10, 95% CI: 1.03–1.18) compared to those in the poorest wealth category. (Table 3). However, protective factors included individuals living in a 3–6-person household—who had an 11% lower risk (RR: 0.89, 95% CI: 0.83–0.96).

**Table 3:** Association between alcohol use and multimorbidity with HIV, and associated factors among participants of a rural South African population of the HAALSI study, enrolled between November 2014 and November 2015 (according to multivariate modified Poisson regression).

Multimorbidity with HIV													
Characteristic	Category	All^d^				Males^e^				Females^f^			
		N^a^	RR^b^	95% CI	*P*-value^c^	N^a^	RR^b^	95% CI	*P*-value^c^	N^a^	RR^b^	95% CI	*P*-value^c^
Alcohol Use	Never	2801	1.00		**< 0.001**	**771**	1.00		**0.036**	2030	1.00		**0.008**
	Ever	1084	**1.05**	**1.02–1.08**		660	**1.05**	**1.00–1.10**		424	**1.06**	**1.02–1.11**	
	Current	1171	0.97	0.94–1.01		912	0.98	0.94–1.03		259	0.97	0.91–1.04	
Respondent Sex	Male	2345	1.00		0.604								
	Female	2714	1.01	0.98–1.04									
Age (years)	40–49	918	1.00		0.171	418	1.00		0.161	500	1.00		0.152
	50–59	1410	1.04	0.99–1.08		624	1.00	0.93–1.08		786	1.05	0.99–1.11	
	60–69	1304	1.05	1.00–1.10		643	1.03	0.96–1.11		661	1.05	0.99–1.11	
	70–79	878	1.03	0.97–1.08		446	1.01	0.93–1.10		432	1.00	0.94–1.07	
	80+	549	1.06	1.00–1.12		214	1.10	1.00–1.20		335	1.00	0.93–1.08	
Body mass index	Underweight	258	0.93	0.87–1.01	**< 0.001**	**188**	**0.90**	**0.82–0.99**	**< 0.001**	**70**	1.04	0.94–1.15	**< 0.001**
	Normal	1719	1			1019	1			700	1		
	Overweight	1328	**1.10**	**1.06–1.13**		611	**1.10**	**1.05–1.16**		717	**1.08**	**1.03–1.13**	
	Obese	1384	**1.12**	**1.09–1.16**		341	**1.15**	**1.09–1.21**		1043	**1.11**	**1.06–1.16**	
Employment status	Employed (part or full time)	805	1.00		0.872	443	1.00		0.981	362	1.00		0.937
	Not Working	3719	0.99	0.95–1.04		1709	1.00	0.94–1.06		2010	0.99	0.94–1.05	
	Homemaker	521	1.00	0.95–1.05		186	**1.01**	**0.92–1.10**		335	0.99	0.93–1.06	
Education	No formal education	2306	1.00		**0.048**	**957**	1.00		0.337	1349	1.00		**0.041**
	Some primary education (1–7 years)	1716	1.01	0.98–1.04		833	1.00	0.95–1.04		883	1.02	0.98–1.05	
	Some secondary education (8–11 years)	574	0.99	0.95–1.04		314	0.96	0.90–1.03		260	1.01	0.95–1.08	
	Secondary or more (12+)	446	**0.92**	**0.87–0.98**		234	0.93	0.85–1.02		212	**0.90**	**0.83–0.98**	
Marital status	Never married	290	1.00		< 0.001	166	1.00		0.044	124	1.00		< 0.001
	Separated or divorced	650	**1.13**	**1.05–1.22**		300	1.10	0.98–1.24		350	**1.14**	**1.03–1.25**	
	Widowed	1540	**1.13**	**1.05–1.21**		276	**1.19**	**1.05–1.34**		1264	1.08	0.99–1.19	
	Currently married	2575	1.07	1.00–1.15		1602	**1.14**	**1.02–1.27**		973	1.01	0.92–1.11	
Number of individuals living in household	Living alone	534	1.00		**0.013**	**330**	1.00		**0.004**				
	Living with one other	538	0.96	0.90–1.02		257	0.93	0.85–1.02					
	Living in 3–6-person household	2438	**0.95**	**0.91–0.99**		1055	**0.89**	**0.83–0.96**					
	Living in 7+ person household	1549	0.99	0.94–1.04		703	0.94	0.87–1.02					
Wealth asset index	Poorest	1046	1.00		0.326	502	1.00		0.024	544	1.00		0.724
	2	1001	0.99	0.95–1.04		455	1.03	0.96–1.10		546	0.97	0.92–1.02	
	3	991	0.99	0.95–1.03		450	1.01	0.95–1.08		541	0.97	0.92–1.02	
	4	1007	1.02	0.98–1.06		457	1.07	1.00–1.14		550	0.99	0.94–1.04	
	Richest	1014	1.03	0.98–1.07		481	**1.10**	**1.03–1.18**		533	0.97	0.92–1.03	

^a^Sample of each exposure category.

^b^Relative risk from modified Poisson regression.

^c^The overall *P*-value for trend across all categories of individual variable.

^d^(Adjusted for: Respondent sex, Age, Body Mass Index, Employment, Education, Marital Status, Number of people in the household, Wealth Index).

^e^(Adjusted for: Age, Body Mass Index, Employment, Education, Marital Status, Wealth Index).

^f^(Adjusted for: Age, Body Mass Index, Employment, Education, Marital Status, Wealth Index).

Significant values are in bold.

### Alcohol use and multimorbidity without HIV

Reported alcohol use was associated with multimorbidity without HIV. Specifically, those reporting ever used alcohol (compared to those who had never used it before) had 5% higher risk of multimorbidity without HIV (RR: 1.05, 95% CI: 1.02–1.09) among all participants and this was similar in males (RR: 1.06, 95%CI: 1.00–1.12) and females (RR: 1.06, 95%CI: 1.01–1.11). The reported current alcohol use was associated with 3% lower risk of multimorbidity without HIV among all participants (RR: 0.97, 95% CI: 0.93–1.01) and among females (RR: 0.95, 95%CI: 0.89–1.03) and 1% lower risk of multimorbidity without HIV in males (RR: 0.99, 95% CI: 0.94–1.05) although not statistically significant.

### Other factors associated with multimorbidity without HIV

#### Among all participants

Others factors associated with multimorbidity without HIV among all participants included age—compared to the reference age category of 40–49 years those aged 50–59 years had 10% higher risk (RR: 1.10, 95% CI: 1.05–1.16), those in the 60–69 year old age category had 16% higher risk (RR: 1.16, 95% CI: 1.10–1.22), those aged 70–79 years had 18% higher risk (RR: 1.18, 95% CI: 1.11–1.25), and 80+ years had a 26% higher risk (RR: 1.26, 95% CI: 1.18–1.34); BMI—those in overweight category had a 15% higher risk (RR: 1.15, 95% CI: 1.11–1.20), and obese individuals had a 20% higher risk (RR: 1.20, 95% CI: 1.16–1.25); marital status—those separated or divorced had a 15% higher risk (RR: 1.15, 95% CI: 1.05–1.26), widowed individuals had an 11% higher risk (RR: 1.11, 95% CI: 1.02–1.22), and those who were currently married had a 12% higher risk (RR: 1.12, 95% CI: 1.03–1.22).

#### Among females

Among females, other factors that were significantly associated with multimorbidity without HIV were: age—those aged 50–59 years had an 11% higher risk (RR: 1.11, 95% CI: 1.04–1.18), 60–69 years-olds (RR: 1.14, 95% CI: 1.07–1.23) and 70–79 year olds (RR: 1.14, 95% CI: 1.06–1.24) both had 14% higher risk, and those aged 80+ years had 18% higher risk (RR: 1.18, 95% CI: 1.09–1.29); BMI—among those in the overweight and obese categories; and marital status: those separated or divorced had 21% higher risk (RR: 1.21, 95% CI: 1.07–1.37) and those who were widowed had a 15% higher risk (RR: 1.15, 95% CI: 1.02–1.29).

#### Among males

Among males, other factors that were significantly associated with multimorbidity without HIV were: age—60–69 years olds had 17% higher risk (RR: 1.17, 95% CI: 1.07–1.27), 70–79 year olds had 19% higher risk (RR: 1.19, 95% CI: 1.09–1.31), and 80+ year olds had 34% higher risk (RR: 1.34, 95% CI: 1.21–1.49); BMI—among those categorized as overweight and obese, specifically, the overweight had 17% higher risk (RR:1.17, 95% CI: 1.11–1.23), and being obese had 22% higher risk (RR: 1.22, 95% CI: 1.15–1.30); and wealth asset index: the richest category had 9% higher risk (RR: 1.09, 95% CI: 1.01–1.18) (Table 4).

**Table 4:** Association between alcohol use and multimorbidity without HIV, and associated factors among participants of a rural South African population of the HAALSI study, enrolled between November 2014 and November 2015 (according to multivariate modified Poisson regression).

Multimorbidity without HIV													
Characteristic	Category	All^d^				Males^e^				Females^f^			
		N^a^	RR^b^	95% CI	*P*-value^c^	N^a^	RR^b^	95% CI	*P*-value^c^	N^a^	RR^b^	95% CI	*P*-value^c^
Alcohol Use	Never	2801	1.00		**< 0.001**	771	1.00		0.052	2030	1.00		**0.013**
	Ever	1084	**1.05**	**1.02–1.09**		660	**1.06**	**1.00–1.12**		424	**1.06**	**1.01–1.11**	
	Current	1171	0.97	0.93–1.01		912	0.99	0.94–1.05		259	0.95	0.89–1.03	
Respondent Sex	Male	2345	1.00		0.218								
	Female	2714	1.03	0.99–1.06									
Age (years)	40–49	918	1.00		**< 0.001**	418	1.00		**< 0.001**	500	1.00		**0.001**
	50–59	1410	**1.10**	**1.05–1.16**		624	1.08	0.99–1.17		786	**1.11**	**1.04–1.18**	
	60–69	1304	**1.16**	**1.10–1.22**		643	**1.17**	**1.07–1.27**		661	**1.14**	**1.07–1.23**	
	70–79	878	**1.18**	**1.11–1.25**		446	**1.19**	**1.09–1.31**		432	**1.14**	**1.06–1.24**	
	80+	549	**1.26**	**1.18–1.34**		214	**1.34**	**1.21–1.49**		335	**1.18**	**1.09–1.29**	
Body mass index	Underweight	258	**0.91**	**0.84–0.99**	**< 0.001**	188	**0.87**	**0.78–0.97**	**< 0.001**	70	1.03	0.91–1.16	**< 0.001**
	Normal	1719	1			1019	1			700	1		
	Overweight	1328	**1.15**	**1.11–1.20**		611	**1.17**	**1.11–1.23**		717	**1.13**	**1.07–1.19**	
	Obese	1384	**1.20**	**1.16–1.25**		341	**1.22**	**1.15–1.30**		1043	**1.18**	**1.13–1.25**	
Employment status	Employed (part or full time)	805	1.00		0.708	443	1.00		0.523	362	1.00		0.93
	Not Working	3719	1.01	0.96–1.06		1709	1.02	0.95–1.09		2010	0.99	0.93–1.06	
	Homemaker	521	1.02	0.96–1.09		186	1.06	0.96–1.16		335	1.00	0.92–1.08	
Education	No formal education	2306	1.00		0.218	957	1.00		0.705	1349	1.00		0.168
	Some primary education (1–7 years)	1716	1.02	0.99–1.05		833	1.02	0.97–1.08		883	1.02	0.98–1.06	
	Some secondary education (8–11 years)	574	0.99	0.93–1.04		314	0.98	0.91–1.06		260	0.99	0.92–1.06	
	Secondary or more (12+)	446	0.96	0.89–1.02		234	0.99	0.90–1.10		212	0.92	0.83–1.01	
Marital status	Never married	290	1.00		**0.029**	166	1.00		0.621	124	1.00		**0.016**
	Separated or divorced	650	**1.15**	**1.05–1.26**		300	1.07	0.93–1.22		350	**1.21**	**1.07–1.37**	
	Widowed	1540	**1.11**	**1.02–1.22**		276	0.98	0.94–1.24		1264	**1.15**	**1.02–1.29**	
	Currently married	2575	**1.12**	**1.03–1.22**		1602	0.99	0.96–1.24		973	1.13	1.00–1.28	
Number of individuals living in household	Living alone	534	1.00		**0.006**	330	1.00		**0.008**	204	1.00		0.315
	Living with one other	538	0.96	0.90–1.02		257	0.92	0.83–1.03		281	0.96	0.88–1.05	
	Living in 3–6-person household	2438	0.96	0.91–1.02		1055	0.92	0.84–1.00		1383	0.97	0.90–1.05	
	Living in 7+ person household	1549	1.02	0.96–1.08		703	0.99	0.90–1.09		846	1.01	0.93–1.09	
Wealth asset index	Poorest	1046	1.00		0.184	502	1.00		**0.046**	544	1.00		0.837
	2	1001	1.00	0.96–1.05		455	1.03	0.96–1.12		546	0.98	0.92–1.04	
	3	991	0.97	0.93–1.02		450	0.99	0.92–1.07		541	0.97	0.91–1.03	
	4	1007	1.02	0.97–1.07		457	1.07	0.99–1.15		550	0.99	0.94–1.05	
	Richest	1014	1.03	0.98–1.08		481	**1.09**	**1.01–1.18**		533	0.99	0.92–1.05	

^a^Sample of each exposure category.

^b^Relative risk from modified Poisson regression.

^c^The overall *P*-value for trend across all categories of individual variable.

^d^(Adjusted for: Respondent sex, Age, Body Mass Index, Employment, Education, Marital Status, Number of people in the household, Wealth Index).

^e^(Adjusted for: Age, Body Mass Index, Employment, Education, Marital Status, Number of people in the household, Wealth Index).

^f^(Adjusted for: Age, Body Mass Index, Employment, Education, Marital Status, Number of people in the household, Wealth Index).

Significant values are in bold.

### Sensitivity analyses

We performed a sensitivity analysis to assess the association between alcohol use and multimorbidity using these reported alcohol use frequency categories: “never”, “ever”, “at least once in the last 30 days”, and “at least once a week”. Similar results were obtained for both HIV multimorbidity and multimorbidity without HIV (Supplementary Tables 7 and 8). This was done to determine the impact of alcohol dose frequency on multimorbidity, and findings were similar to the presented alcohol use categories.

## Discussion

We sought to determine the association between reported alcohol use and multimorbidity in a population of individuals aged ≥ 40 years in rural South Africa. In this analysis, we found that reported alcohol use was common with almost half of the population reporting ever used alcohol. Also, the reported alcohol use was modestly associated with HIV multimorbidity and multimorbidity without HIV.

The observed prevalence of reported alcohol use in this study was 44.6%, which is similar to the WHO reported estimate of 43% among those aged 15 years and above in Africa^2,42^. In this study, across all alcohol use categories, males reported higher and more frequent alcohol consumption than females. Alcohol consumption is an activity dominated by males, with a prevalence reported to be 54% in males and 32% in females and mainly attributed to cultural roles of males and females^43^.

The prevalence of multimorbidity with HIV in the study population was higher than that of multimorbidity without HIV (59.6% vs 52.5%) that is partly explained by the interaction of HIV with various NCDs. The overall prevalence of multimorbidity in this study is within the range of the previously reported prevalence of multimorbidity among older adults in South Africa (30–87%) and (0.7–81.3%) in Low- and Middle-Income countries^44,45^.

Reported ever use alcohol was associated with both multimorbidity with and without HIV in this study population (combination of both males and females), as well as only in females. This may have resulted from a visible dose–response of prior alcohol use that was ceased possibly due to efforts made by individuals to manage multimorbidity^25^. Previous studies on alcohol use and multimorbidity reported that alcohol use was associated with multimorbidity^11,25,46^ and NCDs^13,14,47^, especially in the elderly^1,11,46,48,49^ that could be mainly due to the toxic biochemical effects of alcohol^25^.

The association between reported alcohol use and both multimorbidity with and without HIV in males was not statistically significant. Other studies from high income countries reported a significant association of alcohol on NCDs in males and slightly beneficial for females—owing to the beneficial effect of light to moderate alcohol consumption on both diabetes and ischaemic disease^47^. These contradictions raise concerns to the accuracy of reporting of alcohol use in our study population pointing towards the likelihood of underreporting due to social desirability^50^.

Although non-significant, a more protective association was observed across the entire population of individuals who used alcohol in the last 30 days and both multimorbidity with and without HIV. This could be due to underreporting of alcohol use^50^.

Findings of this study should be interpreted with caution considering the following limitations: social desirability bias caused by underreporting of alcohol consumption patterns commonly occurs in multimorbid individuals—which, although uncertain, may have been the case in our study. If this was the case, this may have resulted in the underreporting of “ever” alcohol use by participants who were told to stop alcohol consumption due to the development of multimorbidity. These incidences of underreporting may have either biased the results towards the null or caused an overestimate of the effect. It is therefore critical to validate reported alcohol consumption using a biomarker especially in chronic diseases management settings. The cross-sectional nature of this study could not allow us to determine the directionality of causation between alcohol use and multimorbidity.

Overall, alcohol use is a significant problem in rural South Africa and globally. Alcohol use has been associated with chronic conditions and odds of having more than one chronic condition at a time (multimorbidity) in rural South Africa and elsewhere in Africa. Despite that, there has been no coordinated alcohol intervention response, with fragmented strategies being implemented across different governmental levels and departments^51^. Two scoping reviews reported on the lack of individual level interventions and integration of such interventions in primary health care settings in sub-Saharan Africa^9,10^.

## Conclusion

Reported alcohol use and multimorbidity were common among adults in rural South Africa. Ever used alcohol was associated with both multimorbidity with and without HIV. Current alcohol use was not associated with multimorbidity potentially due to underreporting because of social desirability. There is an urgent need to integrate alcohol interventions in the management of NCDs and multimorbidity and such interventions should include an objective assessment of alcohol consumption.

## Supplementary Information

Supplementary Tables.

## Data Availability

Data are available in a public, open access repository. Any additional data requests could be directed to chodziwadziwa.kabudula@wits.ac.za. The HAALSI baseline data are publicly available at the Harvard Centre for Population and Development Studies (HCPDS) programme website https://haalsi.org/data.

## Abbreviations

AUD: Alcohol use disorder
BMI: Body mass index
CES-D: Centre for epidemiological studies-depression
CI: Confidence intervals
CWK: Chodziwadziwa. W. Kabudula
DALYs: Disability-adjusted life years
FXG: Francesc Xavier Gómez-Olivé
HAALSI: The Health and Ageing in Africa—a longitudinal Study in an INDEPTH community
HCPDS: Harvard Centre for Population and Development Studies
HDSS: Health and Demographic Surveillance Systems
ICPSR: Inter-University Consortium for Political and Social Research
JMF: Joel Msafiri Francis
MGM: Mafuno G. Mpinganjira
NCDs: Non-communicable diseases
NIA: National Institute on Aging
NIH: National Institutes of Health
PHC: Primary health care
RR: Risk ratio
SBIRT: Screening, brief interventions, and referral for alcohol treatment
SDG: Sustainable development goal
SSA: Sub-Saharan Africa
ST: Stephen Tollman
TC: Tobias Chirwa
WHO: World Health Organisation

## References

[1] Nunes BP, Batista SRR, de Andrade FB, de Souza Junior PRB, Lima-Costa MF, Facchini LA (2018). Multimorbidity: The Brazilian longitudinal study of aging (ELSI-Brazil). Rev. Saúde Públ..

[2] World Health Organization. Global status report on alcohol and health 2018. *World Health Organization* (2019).

[3] Tan CH, Denny CH, Cheal NE, Sniezek JE, Kanny D (2015). Alcohol use and binge drinking among women of childbearing age—United States, 2011–2013. Morb. Mortal. Wkly. Rep..

[4] Baum MK, Rafie C, Lai S, Sales S, Page JB, Campa A (2010). Alcohol use accelerates HIV disease progression. AIDS Res. Hum. Retrovir..

[5] Wu ES, Metzger DS, Lynch KG, Douglas SD (2011). Association between alcohol use and HIV viral load. J. Acquir. Immune Defic. Syndr..

[6] Parry CD, Kekwaletswe C, Shuper PA, Nkosi S, Myers BJ, Morojele NK (2016). Heavy alcohol use in patients on highly active antiretroviral therapy: What responses are needed?. SAMJ S. Afr. Med. J..

[7] Kader R, Seedat S, Govender R, Koch J, Parry C (2014). Hazardous and harmful use of alcohol and/or other drugs and health status among South African patients attending HIV clinics. AIDS Behav..

[8] Peltzer K, Matseke G, Azwihangwisi M, Babor T (2008). Evaluation of alcohol screening and brief intervention in routine practice of primary care nurses in Vhembe district, South Africa. Croat. Med. J..

[9] Mushi D, Francis JM, Moshiro C, Hanlon C, Teferra S (2022). Integration of alcohol use disorder interventions in general health care settings in sub-Saharan Africa: A scoping review. Front. Psychiatry.

[10] Francis JM, Cook S, Morojele NK, Swahn MH (2020). Rarity and limited geographical coverage of individual level alcohol interventions in sub Saharan Africa: Findings from a scoping review. J. Subst. Use.

[11] de Melo LA, de Castro Braga L, Leite FPP, Bittar BF, de Figueirêdo Oséas JM, de Lima KC (2019). Factors associated with multimorbidity in the elderly: An integrative literature review. Rev. Bras. Geriatr. Gerontol..

[12] Keetile M, Navaneetham K, Letamo G (2020). Prevalence and correlates of multimorbidity among adults in Botswana: A cross-sectional study. PLoS ONE.

[13] Rehm J, Hasan OS, Imtiaz S, Probst C, Roerecke M, Shield K (2018). Alcohol and noncommunicable disease risk. Curr. Addict. Rep..

[14] Hurst JR, Agarwal G, van Boven JF, Daivadanam M, Gould GS, Huang EW-C (2020). Critical review of multimorbidity outcome measures suitable for low-income and middle-income country settings: Perspectives from the Global Alliance for Chronic Diseases (GACD) researchers. BMJ Open.

[15] Shi A, Tao Z, Wei P, Zhao J (2016). Epidemiological aspects of heart diseases. Exp. Ther. Med..

[16] Day E, Rudd JH (2019). Alcohol use disorders and the heart. Addiction.

[17] Briasoulis A, Agarwal V, Messerli FH (2012). Alcohol consumption and the risk of hypertension in men and women: A systematic review and meta-analysis. J. Clin. Hypertens..

[18] Fernandez-Sola J (2015). Cardiovascular risks and benefits of moderate and heavy alcohol consumption. Nat. Rev. Cardiol..

[19] Rehm J, Roerecke M (2017). Cardiovascular effects of alcohol consumption. Trends Cardiovasc. Med..

[20] Polsky S, Akturk HK (2017). Alcohol consumption, diabetes risk, and cardiovascular disease within diabetes. Curr. Diabetes Rep..

[21] Rehm J (2011). The risks associated with alcohol use and alcoholism. Alcohol Res. Health.

[22] Lyu H, Tang H, Liang Y, Huang S, Wang Y, Huang W (2022). Alcohol consumption and risk of liver fibrosis in people living with HIV: A systematic review and meta-analysis. Front. Immunol..

[23] Llamosas-Falcon L, Probst C, Buckley C, Jiang H, Lasserre AM, Puka K (2022). Sex-specific association between alcohol consumption and liver cirrhosis: An updated systematic review and meta-analysis. Front. Gastroenterol. (Lausanne).

[24] Amonker S, Houshmand A, Hinkson A, Rowe I, Parker R (2023). Prevalence of alcohol-associated liver disease: A systematic review and meta-analysis. Hepatol. Commun..

[25] Rehm, J. *et al.* Alcohol. *Disease Control Priorities in Developing Countries* 2nd edn (eds Jamison, D. T. *et al.*) https://www.ncbi.nlm.nih.gov/books/NBK11720/ (The International Bank for Reconstruction and Development/The World Bank, 2006). 21250309

[26] Gómez-Olivé FX, Montana L, Wagner RG, Kabudula CW, Rohr JK, Kahn K (2018). Cohort profile: Health and ageing in Africa: A longitudinal study of an indepth community in South Africa (HAALSI). Int. J. Epidemiol..

[27] Vyas S, Kumaranayake L (2006). Constructing socio-economic status indices: How to use principal components analysis. Health Policy Plan..

[28] World Health Organization. Obesity and overweight2021 2 January 2023. Available from: https://www.who.int/news-room/fact-sheets/detail/obesity-and-overweight.

[29] Chang AY, Gómez-Olivé FX, Payne C, Rohr JK, Manne-Goehler J, Wade AN (2019). Chronic multimorbidity among older adults in rural South Africa. BMJ Glob. Health.

[30] Chobanian AV, Bakris GL, Black HR, Cushman WC, Green LA, Izzo JL (2003). Seventh report of the joint national committee on prevention, detection, evaluation, and treatment of high blood pressure. Hpertension.

[31] American Diabetes Association (2020). 2 Classification and diagnosis of diabetes: Standards of Medical Care in Diabetes—2020. Diabetes Care.

[32] Shisana, O., Labadarios, D., Rehle, T., Simbayi, L., Zuma, K., Dhansay, A. *et al*. The South African National Health and Nutrition Examination Survey, 2012: SANHANES-1: The health and nutritional status of the nation (2014).

[33] Rose G, McCartney P, Reid D (1977). Self-administration of a questionnaire on chest pain and intermittent claudication. J. Epidemiol. Community Health.

[34] Ehrlich R, White N, Norman R, Laubscher R, Steyn K, Lombard C (2004). Predictors of chronic bronchitis in South African adults. Int. J. Tuberc. Lung Dis..

[35] Radloff LS (1977). The CES-D scale: A self-report depression scale for research in the general population. Appl. Psychol. Meas..

[36] Breslau N, Peterson EL, Kessler RC, Schultz LR (1999). Short screening scale for DSM-IV posttraumatic stress disorder. Am. J. Psychiatry.

[37] Wade AN, Payne CF, Berkman L, Chang A, Gomez-Olive FX, Kabudula C (2021). Multimorbidity and mortality in an older, rural black South African population cohort with high prevalence of HIV findings from the HAALSI study. BMJ Open.

[38] Barnett K, Mercer SW, Norbury M, Watt G, Wyke S, Guthrie B (2012). Epidemiology of multimorbidity and implications for health care, research, and medical education: a cross-sectional study. The Lancet.

[39] Aoms. Multimorbidity: A priority for global health research. *Academy of Medical Sciences* (2018).

[40] Hawkesworth M (1997). Confounding gender. Signs J. Women Cult. Soc..

[41] Miettinen OS, Cook EF (1981). Confounding: essence and detection. Am. J. Epidemiol..

[42] Ayano G, Yohannis K, Abraha M, Duko B (2019). The epidemiology of alcohol consumption in Ethiopia: A systematic review and meta-analysis. Subst. Abuse Treat. Prev. Policy.

[43] White AM (2020). Gender differences in the epidemiology of alcohol use and related harms in the United States. Alcohol Res. Curr. Rev..

[44] Roomaney RA, van Wyk B, Turawa EB, Pillay-van Wyk V (2021). Multimorbidity in South Africa: A systematic review of prevalence studies. BMJ Open.

[45] Asogwa OA, Boateng D, Marza-Florensa A, Peters S, Levitt N, van Olmen J (2022). Multimorbidity of non-communicable diseases in low-income and middle-income countries: A systematic review and meta-analysis. BMJ Open.

[46] Stewart D, McCambridge J (2019). Alcohol complicates multimorbidity in older adults. Br. Med. J. Publ. Gr..

[47] Parry CD, Patra J, Rehm J (2011). Alcohol consumption and non-communicable diseases: Epidemiology and policy implications. Addiction.

[48] Magodoro IM, Esterhuizen TM, Chivese T (2016). A cross-sectional, facility based study of comorbid non-communicable diseases among adults living with HIV infection in Zimbabwe. BMC Res. Notes.

[49] de Almeida MGN, Nascimento-Souza MA, Lima-Costa MF, Peixoto SV (2020). Lifestyle factors and multimorbidity among older adults (ELSI-Brazil). Eur. J. Ageing.

[50] Kypri K, Wilson A, Attia J, Sheeran P, Miller P, McCambridge J (2016). Social desirability bias in the reporting of alcohol consumption: A randomized trial. J. Stud. Alcohol Drugs.

[51] Parry CD (2005). A review of policy-relevant strategies and interventions to address the burden of alcohol on individuals and society in South Africa. Agnes Karll Schwest. Krankenpfl..

