# Supplementary material for: Association of alcohol use and multimorbidity among adults aged 40 years and above in rural South Africa
Source: Sci Rep. 2023 May 14;13:7807. doi: 10.1038/s41598-023-35018-6 (PMC10183457; doi:10.1038/s41598-023-35018-6)
Supplement: Supplementary file 1 — Supplementary Tables. [file 41598_2023_35018_MOESM1_ESM.docx]

Supplementary(S)Tables

STable 1: Sociodemographic factors associated with multimorbidity (with HIV) among the combined population of male and female participants of a rural South African population of the HAALSI study, enrolled between November 2014 - November 2015

|  |  | Multimorbidity with HIV | | | | | | | | |
| --- | --- | --- | --- | --- | --- | --- | --- | --- | --- | --- |
|  |  | Total | | No chronic condition | | 1 chronic condition | | 2 or more chronic conditions | |  |
| Characteristic | Categories | N | %^a^ | N | %^a^ | N | %^a^ | N | %^a^ | P-value^b^ |
|  |  |  |  |  |  |  |  |  |  |  |
| Respondent sex | Male | 2345 | 46.4 | 305 | 53.9 | 728 | 49.2 | 1312 | 43.5 |  |
|  | Female | 2714 | 53.7 | 261 | 46.1 | 751 | 50.8 | 1702 | 56.5 |  |
|  |  |  |  |  |  |  |  |  |  |  |
| Age (years) | 40-49 | 918 | 18.2 | 147 | 26.0 | 278 | 18.8 | 493 | 16.4 | **<0.001** |
|  | 50-59 | 1410 | 27.9 | 165 | 29.2 | 398 | 26.9 | 847 | 28.1 |  |
|  | 60-69 | 1304 | 25.8 | 388 | 19.8 | 388 | 26.2 | 804 | 26.7 |  |
|  | 70-79 | 878 | 17.4 | 94 | 16.6 | 250 | 16.9 | 534 | 17.7 |  |
|  | 80+ | 549 | 10.9 | 48 | 8.5 | 165 | 11.2 | 336 | 11.2 |  |
|  |  |  |  |  |  |  |  |  |  |  |
| Body mass index | Underweight | 258 | 5.5 | 45 | 9.3 | 85 | 6.2 | 128 | 4.5 | **<0.001** |
|  | Normal | 1719 | 36.7 | 232 | 47.9 | 551 | 40.0 | 936 | 33.1 |  |
|  | Overweight | 1328 | 28.3 | 110 | 22.7 | 376 | 27.3 | 842 | 29.8 |  |
|  | Obese | 1384 | 29.5 | 97 | 20.0 | 364 | 26.5 | 923 | 32.6 |  |
|  |  |  |  |  |  |  |  |  |  |  |
| Employment status | Employed (part or full time) | 805 | 16.0 | 108 | 19.2 | 234 | 15.9 | 463 | 15.4 | **0.063** |
|  | Not Working | 3719 | 73.7 | 411 | 73.1 | 1081 | 73.3 | 2227 | 74.0 |  |
|  | Homemaker | 521 | 10.3 | 43 | 7.7 | 160 | 10.9 | 318 | 10.6 |  |
|  |  |  |  |  |  |  |  |  |  |  |
| Education | No formal education | 2306 | 45.7 | 243 | 43.1 | 675 | 45.8 | 1388 | 46.2 | **0.001** |
|  | Some primary education (1-7 years) | 1716 | 34.0 | 175 | 31.0 | 485 | 32.9 | 1056 | 35.2 |  |
|  | Some secondary education (8-11 years) | 574 | 11.4 | 74 | 13.1 | 167 | 11.3 | 333 | 11.1 |  |
|  | Secondary or more (12+) | 446 | 8.9 | 72 | 12.8 | 147 | 10.0 | 227 | 7.6 |  |
|  |  |  |  |  |  |  |  |  |  |  |
| Marital status | Never married | 290 | 5.7 | 52 | 9.2 | 101 | 6.8 | 137 | 4.6 | **<0.001** |
|  | Separated or divorced | 650 | 12.9 | 73 | 12.9 | 177 | 12 | 400 | 13.3 |  |
|  | Widowed | 1540 | 30.5 | 141 | 25.0 | 409 | 27.7 | 990 | 32.9 |  |
|  | Currently married | 2575 | 50.9 | 299 | 52.9 | 791 | 53.5 | 1485 | 49.3 |  |
|  |  |  |  |  |  |  |  |  |  |  |
| Number of individuals living in household | Living alone | 534 | 10.6 | 64 | 11.3 | 144 | 9.7 | 326 | 10.8 | **0.023** |
|  | Living with one other | 538 | 10.6 | 73 | 12.9 | 143 | 9.7 | 322 | 10.7 |  |
|  | Living in 3–6-person household | 2438 | 48.2 | 284 | 50.2 | 745 | 50.4 | 1409 | 46.8 |  |
|  | Living in 7+ person household | 1549 | 30.6 | 145 | 25.6 | 447 | 30.2 | 957 | 31.8 |  |
|  |  |  |  |  |  |  |  |  |  |  |
| Wealth asset index | Poorest | 1046 | 20.7 | 128 | 22.6 | 312 | 21.1 | 606 | 20.1 | 0.533 |
|  | 2 | 1001 | 19.8 | 117 | 20.7 | 294 | 19.9 | 590 | 19.6 |  |
|  | 3 | 991 | 19.6 | 118 | 20.9 | 291 | 19.7 | 582 | 19.3 |  |
|  | 4 | 1007 | 19.9 | 106 | 18.7 | 280 | 18.9 | 621 | 20.6 |  |
|  | Richest | 1014 | 20.0 | 97 | 17.1 | 302 | 20.4 | 615 | 20.4 |  |
|  |  |  |  |  |  |  |  |  |  |  |
| Alcohol use | Never | 2801 | 55.4 | 300 | 53.1 | 800 | 54.2 | 1701 | 56.4 | **<0.001** |
|  | Ever | 1084 | 21.4 | 106 | 18.8 | 285 | 19.3 | 693 | 23.0 |  |
|  | Current | 1171 | 23.6 | 159 | 28.1 | 392 | 26.5 | 620 | 20.6 |  |

^a^ Column percentage

^b^ P-value for trend across all categories of individual variables

STable 2: Sociodemographic factors associated with multimorbidity (with HIV) among the male participants of a rural South African population of the HAALSI study population of HAALSI study, enrolled between November 2014 - November 2015

|  |  | Multimorbidity with HIV | | | | | | | | |
| --- | --- | --- | --- | --- | --- | --- | --- | --- | --- | --- |
|  |  | Total | | No chronic conditions | | 1 chronic condition | | 2 or more chronic conditions | |  |
| Characteristic | Categories | N | %^a^ | N | %^a^ | N | %^a^ | N | %^a^ | P-value^b^ |
|  |  |  |  |  |  |  |  |  |  |  |
| Age (years) | 40-49 | 418 | 17.8 | 75 | 24.6 | 137 | 18.8 | 206 | 15.7 | **<0.001** |
|  | 50-59 | 624 | 26.6 | 100 | 32.8 | 186 | 25.6 | 338 | 25.8 |  |
|  | 60-69 | 643 | 27.4 | 64 | 21.0 | 207 | 28.4 | 372 | 28.4 |  |
|  | 70-79 | 446 | 19.0 | 47 | 15.4 | 139 | 19.1 | 260 | 19.8 |  |
|  | 80+ | 214 | 9.1 | 19 | 6.2 | 59 | 8.1 | 136 | 10.4 |  |
|  |  |  |  |  |  |  |  |  |  |  |
| Body mass index | Underweight | 188 | 8.7 | 40 | 15.8 | 62 | 9.2 | 86 | 7.0 | **<0.001** |
|  | Normal | 1019 | 47.2 | 140 | 55.3 | 350 | 51.7 | 529 | 43.0 |  |
|  | Overweight | 611 | 28.3 | 50 | 19.8 | 178 | 26.3 | 383 | 31.2 |  |
|  | Obese | 341 | 15.8 | 23 | 9.1 | 87 | 12.9 | 231 | 18.8 |  |
|  |  |  |  |  |  |  |  |  |  |  |
| Employment status | Employed (part or full time) | 443 | 19.0 | 64 | 21.2 | 135 | 18.5 | 244 | 18.7 | 0.573 |
|  | Not Working | 1709 | 73.1 | 220 | 72.9 | 530 | 72.8 | 959 | 73.3 |  |
|  | Homemaker | 186 | 8.0 | 18 | 6.0 | 63 | 8.7 | 105 | 8.0 |  |
|  |  |  |  |  |  |  |  |  |  |  |
| Education | No formal education | 957 | 40.9 | 119 | 39.1 | 300 | 41.4 | 538 | 41.1 | 0.451 |
|  | Some primary education (1-7 years) | 833 | 35.6 | 101 | 33.2 | 254 | 35.0 | 478 | 36.5 |  |
|  | Some secondary education (8-11 years) | 314 | 13.4 | 49 | 16.1 | 91 | 12.6 | 174 | 13.3 |  |
|  | Secondary or more (12+) | 234 | 10.0 | 35 | 11.5 | 80 | 11.0 | 119 | 9.1 |  |
|  |  |  |  |  |  |  |  |  |  |  |
| Marital status | Never married | 166 | 7.1 | 36 | 11.8 | 59 | 8.1 | 71 | 5.4 | **<0.001** |
|  | Separated or divorced | 300 | 12.8 | 53 | 17.4 | 93 | 12.8 | 154 | 11.7 |  |
|  | Widowed | 276 | 11.8 | 33 | 10.9 | 70 | 9.6 | 173 | 13.2 |  |
|  | Currently married | 1602 | 68.3 | 182 | 59.9 | 506 | 69.5 | 914 | 70.0 |  |
|  |  |  |  |  |  |  |  |  |  |  |
| Number of individuals living in household | Living alone | 330 | 14.1 | 47 | 15.4 | 95 | 13.1 | 188 | 14.3 | **0.013** |
|  | Living with one other | 257 | 11.0 | 42 | 13.8 | 62 | 8.5 | 153 | 11.7 |  |
|  | Living in 3-6- person household | 1055 | 45.0 | 137 | 44.9 | 361 | 49.6 | 557 | 42.5 |  |
|  | Living in 7+ person household | 703 | 30.0 | 79 | 25.9 | 210 | 28.9 | 414 | 31.6 |  |
|  |  |  |  |  |  |  |  |  |  |  |
| Wealth asset index | Poorest | 502 | 21.4 | 82 | 26.9 | 162 | 22.3 | 258 | 19.7 | **0.018** |
|  | 2 | 455 | 19.4 | 64 | 21.0 | 136 | 18.7 | 255 | 19.4 |  |
|  | 3 | 450 | 19.2 | 63 | 20.7 | 149 | 20.5 | 238 | 18.1 |  |
|  | 4 | 457 | 19.5 | 52 | 17.1 | 139 | 19.1 | 266 | 20.3 |  |
|  | Richest | 481 | 20.5 | 44 | 14.4 | 142 | 19.5 | 295 | 22.5 |  |
|  |  |  |  |  |  |  |  |  |  |  |
| Alcohol use | Never | 771 | 32.9 | 99 | 32.6 | 229 | 31.5 | 443 | 33.8 | **0.002** |
|  | Ever | 660 | 28.2 | 78 | 25.7 | 179 | 24.6 | 403 | 30.7 |  |
|  | Current | 912 | 38.9 | 127 | 41.8 | 319 | 43.9 | 466 | 35.5 |  |

^a^ Column percentage

^b^ P-value for trend across all categories of individual variables

STable 3: Sociodemographic factors associated with multimorbidity (with HIV) among the female participants of a rural South African population of the HAALSI study population of HAALSI study, enrolled between November 2014 - November 2015

|  |  | Multimorbidity with HIV | | | | | | | | |
| --- | --- | --- | --- | --- | --- | --- | --- | --- | --- | --- |
|  |  | Total | | No chronic conditions | | 1 chronic condition | | 2 or more chronic conditions | |  |
| Characteristic | Categories | N | %^a^ | N | %^a^ | N | %^a^ | N | %^a^ | P-value^b^ |
|  |  |  |  |  |  |  |  |  |  |  |
| Age (years) | 40-49 | 500 | 18.4 | 72 | 27.6 | 141 | 18.8 | 287 | 16.9 | **0.002** |
|  | 50-59 | 786 | 29.0 | 65 | 24.9 | 212 | 28.2 | 509 | 29.9 |  |
|  | 60-69 | 661 | 24.4 | 48 | 18.4 | 181 | 24.1 | 432 | 15.9 |  |
|  | 70-79 | 432 | 15.9 | 47 | 18.0 | 111 | 14.8 | 274 | 16.1 |  |
|  | 80+ | 335 | 12.3 | 29 | 11.1 | 106 | 14.1 | 200 | 11.8 |  |
|  |  |  |  |  |  |  |  |  |  |  |
| Body mass index | Underweight | 70 | 2.8 | 5 | 2.2 | 23 | 3.3 | 42 | 2.6 | **0.001** |
|  | Normal | 700 | 27.7 | 92 | 39.8 | 201 | 28.8 | 407 | 25.4 |  |
|  | Overweight | 717 | 28.3 | 60 | 26.0 | 198 | 28.3 | 459 | 28.7 |  |
|  | Obese | 1043 | 41.2 | 74 | 32.0 | 277 | 39.6 | 692 | 43.3 |  |
|  |  |  |  |  |  |  |  |  |  |  |
| Employment status | Employed (part or full time) | 362 | 13.4 | 44 | 16.9 | 99 | 13.3 | 219 | 12.9 | 0.32 |
|  | Not Working | 2010 | 74.3 | 191 | 73.5 | 551 | 73.8 | 1268 | 74.6 |  |
|  | Homemaker | 335 | 12.4 | 25 | 9.6 | 97 | 13.0 | 213 | 12.5 |  |
|  |  |  |  |  |  |  |  |  |  |  |
| Education | No formal education | 1349 | 49.9 | 124 | 47.7 | 375 | 50.1 | 850 | 50.2 | **0.001** |
|  | Some primary education (1-7 years) | 883 | 32.7 | 74 | 28.5 | 231 | 30.8 | 578 | 34.1 |  |
|  | Some secondary education (8-11 years) | 260 | 9.6 | 25 | 9.6 | 76 | 19.2 | 159 | 9.4 |  |
|  | Secondary or more (12+) | 212 | 7.8 | 37 | 14.2 | 67 | 9.0 | 108 | 6.4 |  |
|  |  |  |  |  |  |  |  |  |  |  |
| Marital status | Never married | 124 | 4.6 | 16 | 6.1 | 42 | 5.6 | 66 | 3.9 | **<0.001** |
|  | Separated or divorced | 350 | 12.9 | 20 | 7.7 | 84 | 11.2 | 246 | 14.5 |  |
|  | Widowed | 1264 | 46.6 | 108 | 41.4 | 339 | 45.2 | 817 | 48.1 |  |
|  | Currently married | 973 | 35.9 | 117 | 44.8 | 285 | 38 | 571 | 33.6 |  |
|  |  |  |  |  |  |  |  |  |  |  |
| Number of individuals living in household | Living alone | 204 | 7.5 | 17 | 6.5 | 49 | 6.5 | 138 | 8.1 | 0.23 |
|  | Living with one other | 281 | 10.4 | 31 | 11.9 | 81 | 10.8 | 169 | 9.9 |  |
|  | Living in 3-6- person household | 1383 | 51 | 147 | 56.3 | 384 | 51.1 | 852 | 50.1 |  |
|  | Living in 7+ person household | 846 | 31.2 | 66 | 25.3 | 237 | 31.6 | 543 | 31.9 |  |
|  |  |  |  |  |  |  |  |  |  |  |
| Wealth asset index | Poorest | 544 | 20 | 46 | 17.6 | 150 | 20 | 348 | 20.5 | 0.77 |
|  | 2 | 546 | 20.1 | 53 | 20.3 | 158 | 21 | 335 | 19.7 |  |
|  | 3 | 541 | 19.9 | 55 | 21.1 | 142 | 18.9 | 344 | 20.2 |  |
|  | 4 | 550 | 20.3 | 54 | 20.7 | 141 | 18.8 | 355 | 20.9 |  |
|  | Richest | 533 | 19.6 | 53 | 20.3 | 160 | 21.3 | 320 | 18.8 |  |
|  |  |  |  |  |  |  |  |  |  |  |
| Alcohol use | Never | 2030 | 74.8 | 201 | 77.0 | 571 | 76.1 | 1258 | 73.9 | **0.036** |
|  | Ever | 424 | 15.6 | 28 | 10.7 | 106 | 14.1 | 290 | 17.0 |  |
|  | Current | 259 | 9.6 | 32 | 12.3 | 73 | 9.7 | 154 | 9.1 |  |

^a^ Column percentage

^b^ P-value for trend across all categories of individual variables

STable 4: Sociodemographic factors associated with multimorbidity (without HIV) among the combined population of male and female participants of a rural South African population of the HAALSI study, enrolled between November 2014 - November 2015

|  |  | Multimorbidity without HIV | | | | | | | | |
| --- | --- | --- | --- | --- | --- | --- | --- | --- | --- | --- |
|  |  | Total | | No chronic conditions | | 1 chronic condition | | 2 or more chronic conditions | |  |
| Characteristic | Categories | N | %^a^ | N | %^a^ | N | %^a^ | N | %^a^ | P-value^b^ |
|  |  |  |  |  |  |  |  |  |  |  |
| Respondent Sex | Male | 2345 | 46.4 | 384 | 54.2 | 827 | 48.9 | 1134 | 42.7 | **<0.001** |
|  | Female | 2714 | 53.4 | 325 | 45.8 | 866 | 51.2 | 1523 | 47.3 |  |
|  |  |  |  |  |  |  |  |  |  |  |
| Age (years) | 40-49 | 918 | 18.2 | 201 | 28.4 | 346 | 20.4 | 371 | 14.0 | **<0.001** |
|  | 50-59 | 1410 | 27.9 | 216 | 30.5 | 472 | 27.9 | 722 | 27.2 |  |
|  | 60-69 | 1304 | 25.8 | 141 | 19.9 | 435 | 25.7 | 728 | 27.4 |  |
|  | 70-79 | 878 | 17.4 | 103 | 14.5 | 270 | 16.0 | 505 | 19.0 |  |
|  | 80+ | 549 | 10.9 | 48 | 6.8 | 170 | 10.0 | 331 | 12.5 |  |
|  |  |  |  |  |  |  |  |  |  |  |
| Body mass index | Underweight | 258 | 5.5 | 58 | 9.3 | 102 | 6.5 | 98 | 4.0 | **<0.001** |
|  | Normal | 1719 | 36.7 | 304 | 48.7 | 654 | 41.3 | 761 | 30.7 |  |
|  | Overweight | 1328 | 28.3 | 145 | 23.2 | 416 | 26.3 | 767 | 30.9 |  |
|  | Obese | 1384 | 29.5 | 117 | 18.8 | 410 | 25.9 | 857 | 34.5 |  |
|  |  |  |  |  |  |  |  |  |  |  |
| Employment status | Employed (part or full time) | 805 | 16.0 | 143 | 20.3 | 286 | 17.0 | 376 | 14.2 | **<0.001** |
|  | Not Working | 3719 | 73.7 | 510 | 72.3 | 1217 | 72.1 | 1992 | 75.1 |  |
|  | Homemaker | 521 | 10.3 | 52 | 7.4 | 184 | 10.9 | 285 | 10.7 |  |
|  |  |  |  |  |  |  |  |  |  |  |
| Education | No formal education | 2306 | 45.7 | 296 | 41.9 | 768 | 45.5 | 1242 | 46.9 | **<0.001** |
|  | Some primary education (1-7 years) | 1716 | 34.0 | 224 | 31.7 | 549 | 32.5 | 943 | 35.6 |  |
|  | Some secondary education (8-11 years) | 574 | 11.4 | 96 | 13.6 | 211 | 12.5 | 267 | 10.1 |  |
|  | Secondary or more (12+) | 446 | 8.9 | 90 | 12.8 | 160 | 9.5 | 196 | 7.4 |  |
|  |  |  |  |  |  |  |  |  |  |  |
| Marital status | Never married | 290 | 5.7 | 71 | 10.0 | 113 | 6.7 | 106 | 4.0 | **<0.001** |
|  | Separated or divorced | 650 | 12.9 | 97 | 13.7 | 220 | 13.0 | 333 | 12.5 |  |
|  | Widowed | 1540 | 30.5 | 176 | 24.9 | 494 | 29.2 | 870 | 32.8 |  |
|  | Currently married | 2575 | 50.9 | 364 | 51.4 | 865 | 51.1 | 1346 | 50.7 |  |
|  |  |  |  |  |  |  |  |  |  |  |
| Number of individuals living in household | Living alone | 534 | 10.6 | 85 | 12.0 | 177 | 10.5 | 272 | 10.2 | **0.001** |
|  | Living with one other | 538 | 10.6 | 92 | 13.0 | 166 | 9.8 | 280 | 10.5 |  |
|  | Living in 3–6-person household | 2438 | 48.2 | 365 | 51.5 | 821 | 48.5 | 1252 | 47.1 |  |
|  | Living in 7+ person household | 1549 | 30.6 | 167 | 23.6 | 529 | 31.3 | 853 | 32.1 |  |
|  |  |  |  |  |  |  |  |  |  |  |
| Wealth asset index | Poorest | 1046 | 20.7 | 165 | 23.3 | 362 | 21.4 | 519 | 19.5 | 0.055 |
|  | 2 | 1001 | 19.8 | 142 | 20.0 | 342 | 20.2 | 517 | 19.5 |  |
|  | 3 | 991 | 19.6 | 154 | 21.7 | 330 | 19.5 | 507 | 19.1 |  |
|  | 4 | 1007 | 19.9 | 131 | 18.5 | 321 | 19.0 | 555 | 20.9 |  |
|  | Richest | 1014 | 20.0 | 117 | 16.5 | 338 | 20.0 | 559 | 21.0 |  |
|  |  |  |  |  |  |  |  |  |  |  |
| Alcohol use | Never | 2801 | 55.4 | 376 | 53.1 | 918 | 54.3 | 1507 | 56.7 | **<0.001** |
|  | Ever | 1084 | 21.4 | 133 | 18.8 | 329 | 19.5 | 622 | 23.4 |  |
|  | Current | 1171 | 23.2 | 199 | 28.1 | 444 | 26.3 | 528 | 19.9 |  |

^a^ Column percentage

^b^ P-value for trend across all categories of individual variables

STable 5: Sociodemographic factors associated with multimorbidity (without HIV) among male participants of a rural South African population of the HAALSI study population of HAALSI study, enrolled between November 2014 - November 2015

|  |  | Multimorbidity without HIV | | | | | | | | |
| --- | --- | --- | --- | --- | --- | --- | --- | --- | --- | --- |
|  |  | Total | | No chronic conditions | | 1 chronic condition | | 2 or more chronic conditions | |  |
| Characteristic | Categories | N | %^a^ | N | %^a^ | N | %^a^ | N | %^a^ | P-value^b^ |
|  |  |  |  |  |  |  |  |  |  |  |
| Age (years) | 40-49 | 418 | 17.8 | 104 | 27.1 | 164 | 19.8 | 150 | 13.2 | **<0.001** |
|  | 50-59 | 624 | 26.6 | 125 | 32.6 | 225 | 27.2 | 274 | 24.2 |  |
|  | 60-69 | 643 | 27.4 | 83 | 21.6 | 226 | 27.3 | 334 | 29.5 |  |
|  | 70-79 | 446 | 19.0 | 53 | 13.8 | 149 | 18.0 | 244 | 21.5 |  |
|  | 80+ | 214 | 9.1 | 19 | 5.0 | 63 | 7.6 | 132 | 11.6 |  |
|  |  |  |  |  |  |  |  |  |  |  |
| Body mass index | Underweight | 188 | 8.7 | 51 | 15.5 | 72 | 9.4 | 65 | 6.1 | **<0.001** |
|  | Normal | 1019 | 47.2 | 186 | 56.5 | 404 | 52.5 | 429 | 40.4 |  |
|  | Overweight | 611 | 28.3 | 63 | 19.2 | 195 | 25.4 | 353 | 33.3 |  |
|  | Obese | 341 | 15.8 | 29 | 8.8 | 98 | 12.7 | 214 | 20.2 |  |
|  |  |  |  |  |  |  |  |  |  |  |
| Employment status | Employed (part or full time) | 443 | 19.0 | 84 | 22.1 | 168 | 20.4 | 191 | 16.9 | **0.064** |
|  | Not Working | 1709 | 73.1 | 275 | 72.2 | 589 | 71.4 | 845 | 74.7 |  |
|  | Homemaker | 186 | 8.0 | 22 | 5.8 | 68 | 8.2 | 96 | 8.5 |  |
|  |  |  |  |  |  |  |  |  |  |  |
| Education | No formal education | 957 | 40.9 | 150 | 39.3 | 340 | 41.3 | 467 | 41.3 | 0.376 |
|  | Some primary education (1-7 years) | 833 | 35.6 | 126 | 33.0 | 288 | 35.0 | 419 | 37.0 |  |
|  | Some secondary education (8-11 years) | 314 | 13.3 | 61 | 16.0 | 110 | 13.4 | 143 | 12.6 |  |
|  | Secondary or more (12+) | 234 | 10.0 | 45 | 11.8 | 86 | 10.4 | 103 | 9.1 |  |
|  |  |  |  |  |  |  |  |  |  |  |
| Marital status | Never married | 166 | 7.1 | 44 | 11.5 | 65 | 7.9 | 57 | 5.0 | **<0.001** |
|  | Separated or divorced | 300 | 12.8 | 66 | 17.2 | 111 | 13.4 | 123 | 10.9 |  |
|  | Widowed | 276 | 11.8 | 43 | 11.2 | 94 | 11.4 | 139 | 12.3 |  |
|  | Currently married | 1602 | 68.3 | 230 | 60.1 | 557 | 67.4 | 815 | 71.9 |  |
|  |  |  |  |  |  |  |  |  |  |  |
| Number of individuals living in household | Living alone | 330 | 14.1 | 61 | 15.9 | 119 | 14.4 | 150 | 13.2 | **0.006** |
|  | Living with one other | 257 | 11.0 | 54 | 14.1 | 74 | 9.0 | 129 | 11.4 |  |
|  | Living in 3-6-person household | 1055 | 45.0 | 180 | 46.9 | 387 | 46.8 | 488 | 42.0 |  |
|  | Living in 7+ person household | 703 | 30.0 | 89 | 23.2 | 247 | 29.9 | 367 | 32.4 |  |
|  |  |  |  |  |  |  |  |  |  |  |
| Wealth asset index | Poorest | 502 | 21.4 | 98 | 25.5 | 190 | 23.0 | 214 | 18.9 | **0.002** |
|  | 2 | 455 | 19.4 | 76 | 17.8 | 160 | 19.4 | 219 | 19.3 |  |
|  | 3 | 450 | 19.2 | 85 | 22.1 | 164 | 19.8 | 201 | 17.7 |  |
|  | 4 | 457 | 19.5 | 69 | 18.0 | 154 | 18.6 | 234 | 20.6 |  |
|  | Richest | 481 | 20.5 | 56 | 14.6 | 159 | 19.2 | 266 | 23.5 |  |
|  |  |  |  |  |  |  |  |  |  |  |
| Alcohol use | Never | 771 | 32.9 | 128 | 33.4 | 258 | 31.2 | 385 | 34.0 | **<0.001** |
|  | Ever | 660 | 28.2 | 98 | 25.6 | 205 | 24.8 | 357 | 31.5 |  |
|  | Current | 912 | 38.9 | 157 | 41.0 | 363 | 44.0 | 392 | 34.6 |  |

^a^ Column percentage

^b^ P-value for trend across all categories of individual variables

STable 6: Sociodemographic factors associated with multimorbidity (without HIV) among female participants of a rural South African population of the HAALSI study population of HAALSI study, enrolled between November 2014 - November 2015

|  |  | Multimorbidity without HIV | | | | | | | | |
| --- | --- | --- | --- | --- | --- | --- | --- | --- | --- | --- |
|  |  | Total | | No chronic conditions | | 1 chronic condition | | 2 or more chronic conditions | |  |
| Characteristic | Categories | N | %^a^ | N | %^a^ | N | %^a^ | N | %^a^ | P-value^b^ |
|  |  |  |  |  |  |  |  |  |  |  |
| Age (years) | 40-49 | 500 | 18.4 | 97 | 29.9 | 182 | 21.0 | 221 | 14.5 | **<0.001** |
|  | 50-59 | 786 | 29.0 | 91 | 28.0 | 247 | 28.5 | 448 | 29.4 |  |
|  | 60-69 | 661 | 24.4 | 58 | 17.9 | 209 | 24.1 | 394 | 25.9 |  |
|  | 70-79 | 432 | 15.9 | 50 | 15.4 | 121 | 14.0 | 261 | 17.1 |  |
|  | 80+ | 335 | 12.3 | 29 | 8.9 | 107 | 12.4 | 199 | 13.1 |  |
|  |  |  |  |  |  |  |  |  |  |  |
| Body mass index | Underweight | 70 | 2.8 | 7 | 2.4 | 30 | 3.7 | 33 | 2.3 | **<0.001** |
|  | Normal | 700 | 27.7 | 118 | 40.0 | 250 | 30.8 | 332 | 23.4 |  |
|  | Overweight | 717 | 28.3 | 82 | 27.8 | 221 | 27.2 | 414 | 29.1 |  |
|  | Obese | 1043 | 41.2 | 88 | 29.8 | 312 | 38.4 | 643 | 54.2 |  |
|  |  |  |  |  |  |  |  |  |  |  |
| Employment status | Employed (part or full time) | 362 | 13.4 | 59 | 18.2 | 118 | 13.7 | 185 | 12.2 | **0.023** |
|  | Not Working | 2010 | 74.3 | 235 | 72.5 | 628 | 72.9 | 1147 | 75.4 |  |
|  | Homemaker | 335 | 12.4 | 30 | 9.3 | 116 | 13.5 | 189 | 12.4 |  |
|  |  |  |  |  |  |  |  |  |  |  |
| Education | No formal education | 1349 | 49.9 | 146 | 45.1 | 428 | 49.5 | 775 | 51.1 | **<0.001** |
|  | Some primary education (1-7 years) | 883 | 32.7 | 98 | 30.3 | 261 | 30.2 | 524 | 34.6 |  |
|  | Some secondary education (8-11 years) | 260 | 9.6 | 35 | 10.8 | 101 | 11.7 | 124 | 8.2 |  |
|  | Secondary or more (12+) | 212 | 7.8 | 45 | 13.9 | 74 | 8.6 | 93 | 6.1 |  |
|  |  |  |  |  |  |  |  |  |  |  |
| Marital status | Never married | 124 | 4.6 | 27 | 8.3 | 48 | 5.6 | 49 | 3.2 | **<0.001** |
|  | Separated or divorced | 350 | 12.9 | 31 | 9.5 | 109 | 12.6 | 210 | 13.8 |  |
|  | Widowed | 1264 | 46.6 | 133 | 40.9 | 400 | 46.2 | 731 | 48.1 |  |
|  | Currently married | 973 | 35.9 | 134 | 41.2 | 308 | 35.6 | 531 | 34.9 |  |
|  |  |  |  |  |  |  |  |  |  |  |
| Number of individuals living in household | Living alone | 204 | 7.5 | 24 | 7.4 | 58 | 6.7 | 122 | 8.0 | **0.091** |
|  | Living with one other | 281 | 10.4 | 38 | 11.7 | 92 | 10.6 | 151 | 9.9 |  |
|  | Living in 3-6-person household | 1383 | 51.0 | 185 | 56.9 | 434 | 50.1 | 764 | 50.2 |  |
|  | Living in 7+ person household | 846 | 31.2 | 78 | 24.0 | 282 | 32.6 | 486 | 31.9 |  |
|  |  |  |  |  |  |  |  |  |  |  |
| Wealth asset index | Poorest | 544 | 20.0 | 67 | 20.6 | 172 | 19.9 | 305 | 20.0 | 0.931 |
|  | 2 | 546 | 20.1 | 66 | 20.3 | 182 | 21.0 | 298 | 19.6 |  |
|  | 3 | 541 | 19.9 | 69 | 21.2 | 166 | 19.2 | 306 | 20.1 |  |
|  | 4 | 550 | 20.3 | 62 | 19.1 | 167 | 19.3 | 321 | 21.1 |  |
|  | Richest | 533 | 19.6 | 61 | 18.8 | 179 | 20.7 | 293 | 19.2 |  |
|  |  |  |  |  |  |  |  |  |  |  |
| Alcohol use | Never | 2030 | 74.8 | 248 | 76.3 | 660 | 76.3 | 1122 | 73.7 | **0.007** |
|  | Ever | 424 | 15.6 | 35 | 10.8 | 124 | 14.3 | 265 | 17.4 |  |
|  | Current | 259 | 9.6 | 42 | 12.9 | 81 | 9.4 | 136 | 8.9 |  |

^a^ Column percentage

^b^ P-value for trend across all categories of individual variables

Table 7: Association between alcohol use and multimorbidity with HIV, and associated factors among participants of a rural South African population of the HAALSI study, enrolled between November 2014 - November 2015 (according to modified Poisson)

| Multimorbidity with HIV | | | | | | | | | | | | | |
| --- | --- | --- | --- | --- | --- | --- | --- | --- | --- | --- | --- | --- | --- |
| Characteristic | Category | All ^d^ | | | | Males ^e^ | | | | Females ^f^ | | | |
|  |  | N^a^ | RR^b^ | 95% CI | P-value^c^ | N^a^ | RR^b^ | 95% CI | P-value^c^ | N^a^ | RR^b^ | 95% CI | P-value^c^ |
|  |  |  |  |  |  |  |  |  |  |  |  |  |  |
| Alcohol Use | Never | 2801 | 1.00 |  | **<0.001** | **771** | 1.00 |  | 0.082 | 2030 | 1.00 |  | **0.012** |
|  | Ever | 1084 | **1.05** | **1.02-1.09** |  | 660 | 1.05 | 1.00-1.10 |  | 424 | **1.06** | **1.02-1.11** |  |
|  | At least once a month | 551 | 0.97 | 0.92-1.01 |  | 392 | 0.98 | 0.92-1.04 |  | 159 | 0.95 | 0.87-1.03 |  |
|  | At least once a week | 619 | 0.98 | 0.93-1.03 |  | 519 | 0.99 | 0.93-1.05 |  | 100 | 1.02 | 0.93-1.12 |  |
|  |  |  |  |  |  |  |  |  |  |  |  |  |  |
| Respondent Sex | Male | 2345 | 1.00 |  | 0.595 |  |  |  |  |  |  |  |  |
|  | Female | 2714 | 1.01 | 0.98-1.04 |  |  |  |  |  |  |  |  |  |
|  |  |  |  |  |  |  |  |  |  |  |  |  |  |
| Age (years) | 40-49 | 918 | 1.00 |  | 0.168 | 418 | 1.00 |  | 0.143 | 500 | 1.00 |  | 0.165 |
|  | 50-59 | 1410 | 1.04 | 0.99-1.08 |  | 624 | 1.00 | 0.93-1.08 |  | 786 | 1.05 | 0.99-1.11 |  |
|  | 60-69 | 1304 | 1.05 | 1.00-1.10 |  | 643 | 1.03 | 0.96-1.11 |  | 661 | 1.05 | 0.99-1.11 |  |
|  | 70-79 | 878 | 1.03 | 0.97-1.08 |  | 446 | 1.01 | 0.94-1.10 |  | 432 | 1.00 | 0.94-1.07 |  |
|  | 80+ | 549 | 1.06 | 1.00-1.12 |  | 214 | 1.10 | 1.00-1.20 |  | 335 | 1.00 | 0.93-1.08 |  |
|  |  |  |  |  |  |  |  |  |  |  |  |  |  |
| Body mass index | Underweight | 258 | 1.00 |  | **<0.001** | 188 | 1.00 |  | **<0.001** | 70 | 1.00 |  | **<0.001** |
|  | Normal | 1719 | 1.07 | 1.00-1.15 |  | 1019 | **1.11** | **1.01-1.22** |  | 700 | 0.97 | 0.87-1.07 |  |
|  | Overweight | 1328 | **1.17** | **1.09-1.26** |  | 611 | **1.22** | **1.11-1.34** |  | 717 | 1.05 | 0.95-1.16 |  |
|  | Obese | 1384 | **1.21** | **1.12-1.30** |  | 341 | **1.28** | **1.15-1.41** |  | 1043 | 1.07 | 0.97-1.18 |  |
|  |  |  |  |  |  |  |  |  |  |  |  |  |  |
| Employment status | Employed (part or full time) | 805 | 1.00 |  | 0.872 | 443 | 1.00 |  | 0.982 | 362 | 1.00 |  | 0.932 |
|  | Not Working | 3719 | 0.99 | 0.95-1.03 |  | 1709 | 1.00 | 0.94-1.06 |  | 2010 | 0.99 | 0.94-1.05 |  |
|  | Homemaker | 521 | 1.00 | 0.95-1.05 |  | 186 | 1.01 | 0.92-1.10 |  | 335 | 0.99 | 0.93-1.06 |  |
|  |  |  |  |  |  |  |  |  |  |  |  |  |  |
| Education | No formal education | 2306 | 1.00 |  | 0.048 | 957 | 1.00 |  | 0.341 | 1249 | 1.00 |  | 0.038 |
|  | Some primary education (1-7 years) | 1716 | 1.01 | 0.98-1.04 |  | 833 | 1.00 | 0.95-1.04 |  | 883 | 1.02 | 0.98-1.06 |  |
|  | Some secondary education (8-11 years) | 574 | 0.99 | 0.95-1.04 |  | 314 | 0.96 | 0.90-1.03 |  | 260 | 1.01 | 0.95-1.08 |  |
|  | Secondary or more (12+) | 446 | **0.92** | **0.87-0.98** |  | 234 | 0.93 | 0.85-1.01 |  | 212 | **0.90** | **0.83-0.98** |  |
|  |  |  |  |  |  |  |  |  |  |  |  |  |  |
| Marital status | Never married | 290 | 1.00 |  | **<0.001** | 166 | 1.00 |  | 0.042 | 124 | 1.00 |  | **<0.001** |
|  | Separated or divorced | 650 | **1.14** | **1.05-1.22** |  | 300 | 1.10 | 0.98-1.24 |  | 350 | **1.14** | **1.03-1.25** |  |
|  | Widowed | 1540 | **1.13** | **1.05-1.21** |  | 276 | **1.19** | **1.05-1.34** |  | 1264 | 1.08 | 0.99-1.19 |  |
|  | Currently married | 2575 | 1.07 | 1.00-1.15 |  | 1602 | **1.14** | **1.02-1.27** |  | 973 | 1.01 | 0.92-1.11 |  |
|  |  |  |  |  |  |  |  |  |  |  |  |  |  |
| Number of individuals living in household | Living alone | 534 | 1.00 |  | **0.014** | 330 | 1.00 |  | **0.004** |  |  |  |  |
|  | Living with one other | 538 | 0.96 | 0.91-1.02 |  | 257 | 0.93 | 0.85-1.02 |  |  |  |  |  |
|  | Living in 3–6-person household | 2438 | 0.95 | 0.91-1.00 |  | 1055 | **0.89** | **0.83-0.96** |  |  |  |  |  |
|  | Living in 7+ person household | 1549 | 0.99 | 0.94-1.04 |  | 703 | 0.94 | 0.87-1.02 |  |  |  |  |  |
|  |  |  |  |  |  |  |  |  |  |  |  |  |  |
| Wealth asset index | Poorest | 1046 | 1.00 |  | 0.323 | 502 | 1.00 |  | **0.025** | 544 | 1.00 |  | 0.738 |
|  | 2 | 1001 | 0.99 | 0.95-1.04 |  | 455 | 1.03 | 0.96-1.10 |  | 546 | 0.97 | 0.92-1.02 |  |
|  | 3 | 991 | 0.99 | 0.95-1.03 |  | 450 | 1.01 | 0.95-1.08 |  | 541 | 0.97 | 0.92-1.02 |  |
|  | 4 | 1007 | 1.02 | 0.98-1.06 |  | 457 | 1.07 | 1.00-1.14 |  | 550 | 0.99 | 0.94-1.04 |  |
|  | Richest | 1014 | 1.03 | 0.98-1.07 |  | 481 | **1.10** | **1.03-18** |  | 533 | 0.97 | 0.92-1.03 |  |

^a^ Sample of each exposure category

^b^ Relative risk from modified Poisson regression

^c^ The overall P-value for trend across all categories of individual variable

^d^ (Adjusted for: Respondent sex, Age, Body Mass Index, Employment, Education, Marital Status, Number of people in the household, Wealth Index)

^e^ (Adjusted for: Age, Body Mass Index, Employment, Education, Marital Status, Wealth Index)

^f^ (Adjusted for: Age, Body Mass Index, Employment, Education, Marital Status, Wealth Index)

STable 8: Association between alcohol use and multimorbidity without HIV, and associated factors among participants of a rural South African population of the HAALSI study, enrolled between November 2014 - November 2015 (according to modified Poisson)

| Multimorbidity without HIV | | | | | | | | | | | | | |
| --- | --- | --- | --- | --- | --- | --- | --- | --- | --- | --- | --- | --- | --- |
| Characteristic | Category | All ^d^ | | | | Males ^e^ | | | | Females ^f^ | | | |
|  |  | N^a^ | RR^b^ | 95% CI | P-value^c^ | N^a^ | RR^b^ | 95% CI | P-value^c^ | N^a^ | RR^b^ | 95% CI | P-value^c^ |
|  |  |  |  |  |  |  |  |  |  |  |  |  |  |
| Alcohol Use | Never | 2801 | 1.00 |  | **0.002** | 771 | 1.00 |  | 0.109 | 2030 | 1.00 |  | **0.018** |
|  | Ever | 1084 | **1.05** | **1.02-1.09** |  | 660 | 1.06 | 1.00-1.12 |  | 424 | **1.06** | **1.01-1.11** |  |
|  | At least once a month | 551 | 0.96 | 0.91-1.01 |  | 392 | 0.99 | 0.92-1.05 |  | 159 | 0.92 | 0.84-1.01 |  |
|  | At least once a week | 619 | 0.98 | 0.93-1.04 |  | 519 | 1.00 | 0.93-1.07 |  | 100 | 1.01 | 0.91-1.12 |  |
|  |  |  |  |  |  |  |  |  |  |  |  |  |  |
| Respondent Sex | Male | 2345 | 1.00 |  | 0.205 |  |  |  |  |  |  |  |  |
|  | Female | 2714 | 1.02 | 0.99-1.06 |  |  |  |  |  |  |  |  |  |
|  |  |  |  |  |  |  |  |  |  |  |  |  |  |
| Age (years) | 40-49 | 918 | 1.00 |  | **<0.001** | 418 | 1.00 |  | **<0.001** | 500 | 1.00 |  | **0.001** |
|  | 50-59 | 1410 | **1.10** | **1.05-1.16** |  | 624 | 1.08 | 0.99-1.17 |  | 786 | **1.11** | **1.04-1.18** |  |
|  | 60-69 | 1304 | **1.16** | **1.10-1.22** |  | 643 | **1.17** | **1.07-1.27** |  | 661 | **1.14** | **1.07-1.22** |  |
|  | 70-79 | 878 | **1.18** | **1.11-1.25** |  | 446 | **1.19** | **1.09-1.31** |  | 432 | **1.14** | **1.05-1.24** |  |
|  | 80+ | 549 | **1.26** | **1.18-1.35** |  | 214 | **1.34** | **1.21-1.49** |  | 335 | **1.18** | **1.09-1.29** |  |
|  |  |  |  |  |  |  |  |  |  |  |  |  |  |
| Body mass index | Underweight | 258 | 1.00 |  | **<0.001** | 188 | 1.00 |  | **<0.001** | 70 | 1.00 |  | **<0.001** |
|  | Normal | 1719 | **1.10** | **1.01-1.20** |  | 1019 | **1.15** | **1.03-1.28** |  | 700 | 0.97 | 0.86-1.10 |  |
|  | Overweight | 1328 | **1.27** | **1.16-1.38** |  | 611 | **1.35** | **1.21-1.51** |  | 717 | 1.10 | 0.98-1.24 |  |
|  | Obese | 1384 | **1.32** | **1.22-1.44** |  | 341 | **1.41** | **1.25-1.58** |  | 1043 | **1.16** | **1.03-1.30** |  |
|  |  |  |  |  |  |  |  |  |  |  |  |  |  |
| Employment status | Employed (part or full time) | 805 | 1.00 |  | 0.695 | 443 | 1.00 |  | 0.524 | 362 | 1.00 |  | 0.911 |
|  | Not Working | 3719 | 1.01 | 0.96-1.05 |  | 1709 | 1.02 | 0.95-1.09 |  | 2010 | 0.99 | 0.93-1.06 |  |
|  | Homemaker | 521 | 1.02 | 0.97-1.09 |  | 186 | 1.06 | 0.96-1.16 |  | 335 | 1.00 | 0.93-1.08 |  |
|  |  |  |  |  |  |  |  |  |  |  |  |  |  |
| Education | No formal education | 2306 | 1.00 |  | 0.218 | 957 | 1.00 |  | 0.708 | 1349 | 1.00 |  | 0.159 |
|  | Some primary education (1-7 years) | 1716 | 1.02 | 0.99-1.05 |  | 833 | 1.02 | 0.97-1.08 |  | 883 | 1.02 | 0.98-1.06 |  |
|  | Some secondary education (8-11 years) | 574 | 0.99 | 0.93-1.04 |  | 314 | 0.98 | 0.91-1.06 |  | 260 | 0.99 | 0.92-1.07 |  |
|  | Secondary or more (12+) | 446 | 0.96 | 0.89-1.03 |  | 234 | 0.99 | 0.90-1.10 |  | 212 | 0.92 | 0.83-1.01 |  |
|  |  |  |  |  |  |  |  |  |  |  |  |  |  |
| Marital status | Never married | 290 | 1.00 |  | **0.029** | 166 | 1.00 |  | 0.625 | 124 | 1.00 |  | **0.014** |
|  | Separated or divorced | 650 | **1.15** | **1.05-1.26** |  | 300 | 1.07 | 0.93-1.22 |  | 350 | **1.21** | **1.07-1.37** |  |
|  | Widowed | 1540 | **1.11** | **1.02-1.22** |  | 276 | 1.08 | 0.94-1.23 |  | 1264 | **1.15** | **1.02-1.29** |  |
|  | Currently married | 2575 | **1.12** | **1.03-1.22** |  | 1602 | 1.09 | 0.96-1.23 |  | 973 | **1.13** | **1.01-1.28** |  |
|  |  |  |  |  |  |  |  |  |  |  |  |  |  |
| Number of individuals living in household | Living alone | 534 | 1.00 |  | **0.006** | 330 | 1.00 |  | **0.008** | 204 | 1.00 |  | 0.307 |
|  | Living with one other | 538 | 0.96 | 0.90-1.03 |  | 257 | 0.92 | 0.83-1.03 |  | 281 | 0.96 | 0.88-1.05 |  |
|  | Living in 3–6-person household | 2438 | 0.96 | 0.91-1.02 |  | 1055 | 0.92 | 0.84-1.00 |  | 1383 | 0.97 | 0.90-1.05 |  |
|  | Living in 7+ person household | 1549 | 1.02 | 0.96-1.08 |  | 703 | 0.99 | 0.90-1.09 |  | 846 | 1.01 | 0.93-1.09 |  |
|  |  |  |  |  |  |  |  |  |  |  |  |  |  |
| Wealth asset index | Poorest | 1046 | 1.00 |  | 0.181 | 502 | 1.00 |  | **0.046** | 544 | 1.00 |  | 0.844 |
|  | 2 | 1001 | 1.00 | 0.96-1.05 |  | 455 | 1.03 | 0.96-1.12 |  | 546 | 0.98 | 0.92-1.04 |  |
|  | 3 | 991 | 0.97 | 0.93-1.02 |  | 450 | 0.99 | 0.92-1.07 |  | 541 | 0.97 | 0.91-1.03 |  |
|  | 4 | 1007 | 1.02 | 0.97-1.07 |  | 457 | 1.07 | 0.99-1.14 |  | 550 | 0.99 | 0.94-1.05 |  |
|  | Richest | 1014 | 1.03 | 0.98-1.08 |  | 481 | **1.09** | **1.01-1.18** |  | 533 | 0.99 | 0.93-1.05 |  |

^a^ Sample of each exposure category

^b^ Relative risk from modified Poisson regression

^c^ The overall P-value for trend across all categories of individual variable

^d^ (Adjusted for: Respondent sex, Age, Body Mass Index, Employment, Education, Marital Status, Number of people in the household, Wealth Index)

^e^ (Adjusted for: Age, Body Mass Index, Employment, Education, Marital Status, Number of people in the household, Wealth Index)

^f^ (Adjusted for: Age, Body Mass Index, Employment, Education, Marital Status, Number of people in the household, Wealth Index)
